# COVID-19 Infection in Cancer Patients: How Can Oncologists Deal With These Patients?

**DOI:** 10.3389/fonc.2020.00734

**Published:** 2020-04-23

**Authors:** Oronzo Brunetti, Afshin Derakhshani, Behzad Baradaran, Antonio Galvano, Antonio Russo, Nicola Silvestris

**Affiliations:** ^1^Medical Oncology Unit IRCCS Istituto Tumori “Giovanni Paolo II” of Bari, University of Bari “Aldo Moro”, Bari, Italy; ^2^Immunology Research Center, Tabriz University of Medical Sciences, Tabriz, Iran; ^3^Department of Surgical, Oncological and Oral Sciences, Section of Medical Oncology, University of Palermo, Palermo, Italy; ^4^Department of Biomedical Sciences and Medical Oncology, University of Bari, Bari, Italy

**Keywords:** COVID-19, cancer, therapy, screening, prognosis

The world is facing a new pandemic caused by a novel beta coronavirus (COVID 19), which causes severe respiratory coronavirus syndrome (SARS-CoV-2). Unfortunately, there are currently no US Food and Drug Administration (FDA) approved medications for the treatment of COVID-19 patients. High mortality rates in frail patients is a notable feature of the virus registered since the onset of COVID-19 pandemic. Above all, elderly patients or those with underlying chronic illnesses and compromised immune system are most at risk (1). Thus, the consideration is that the possible coexistence, in the same individual, of a cancer diagnosis and a COVID-19 infection could generate a synergistic negative prognostic effect.

About the prevalence, the only available data are reported in a retrospective study on 1,590 COVID-19 patients from 575 Chinese hospitals, wherein 1% of them had a history of malignancy (2). Among cancer patients, 25% of them had recently received chemotherapy or surgical treatment, while the others were in follow-up. Multivariate analysis showed that a history of cancer was associated with a higher risk of serious/negative events (OR = 5.399, *P* = 0.003), with a median time in their development of 13 vs. 43 days in non-cancer patients (*P* < 0.0001; hazard ratio = 3.56, 95% confidence interval = 1.65–7.69). Therefore, the diagnosis of cancer was shown to be an important comorbidity associated with a higher rate of intensive care admissions.

Several questions still remain unanswered in this scenario: (1) Do routine screening programs need to continue as usual? (2) What should be the correct management for a positive COVID-19 patient before or after a cycle of chemotherapy, immunotherapy, and/or targeted therapy? (3) What policies are being adopted in every single country to manage oncology departments? (4) How should fragile patients with the advanced disease be treated when they are in areas heavily affected by the virus? (5) What are the ethical and practical implications?

In other words, how can oncologists deal with these patients? Can we find these answers by analyzing the data available in the literature? Unfortunately, it is not possible. At present, the follow-up period from the moment of the COVID-19 pandemic onset is too short to reach definitive conclusions.

According to recent American Cancer Society guidelines for cancer patients during the COVID-19 pandemic, screening programs should be delayed (3).

It should appear to be a reasonable choice during this COVID-19 crisis period to carry out a risk/benefit balance in every patient evaluation, especially when the potential benefit of an oncological intervention in terms of cancer recurrence/overall survival is so small that it doesn't counterbalance the potential risk of death from COVID-19.

Certainly, triage by telephone with the possible identification of symptomatic patients and at the entrance of hospital structures with the monitoring of body temperature and saturation could reduce the probability for health personnel and for the patients themselves to be exposed to the risk of contagion.

Cancer thoracic/abdominal surgery sometimes requires recovery in intensive care units. How should these needs be managed need in emergency situations? On the other hand, the risk is that a localized disease may become metastatic over time.

Patients in treatment for neoadjuvant/adjuvant treatments should continue their treatments, and, if it is possible should also receive longer intervals between a cycle, longer treatments, and the avoiding of weekly infusions, considering every specific drug schedule. For advanced cancer patients, it should be decided on a case-by-case basis, taking into consideration the relationship between health risks and benefits, whether to delay or suspend ongoing treatments for a period, miming the already known “drug holiday.” So, the considerable risk is that patients will survive COVID-19, but then endanger their lives due to the absence of sufficient cancer care.

Last but not least, the alterations in the mood of these patients cannot be underestimated, just like this aspect cannot be underestimated among clinicians and paramedics working in oncology departments.

The follow up of patients without evidence of recurrence should be postponed or carried out through “remote visits,” through telephone, email, or other telemedicine tools, reserving the possibility to access the cancer center only in selected cases.

It should not be suggested that accompanying persons/family members/caregivers of cancer patients should stay in waiting rooms, or should access rooms where anti-cancer therapy and outpatient clinics are administered. It is advisable to avoid visits to patients during their hospitalization, only suggesting the presence of a single family member/companion, after specific authorization, only for a limited time.

As mentioned above, particular attention should be reserved for older cancer patients, due to their comorbidities.

And what is to be decided regarding oncological research? Cancer Research UK proposes to stop recruiting patients in several clinical trials with two aims: to reduce patient overflow and to dedicate time and financial resources to patients affected by COVID-19 looking for clinical research treatment (4).

More recently, based on a consensus of experts, ASCO (5) recommended several suggestions including the use of proper hand washing, hygiene, and minimizing exposure to sick contacts with the use of masks for patients, indicating a comprehensive evaluation in those with fever or respiratory symptoms. Moreover, ASCO did not make a recommendation with respect to COVID-19 testing in patients with cancer. Regarding cancer patients affected by COVID-19, treatment should not be reinitiated until symptoms of COVID-19 have resolved. Regarding all cancer patients, maintenance chemotherapy may be stopped, if possible intravenous chemotherapy should be orally administrated less frequently at clinics, and decisions on chemotherapy treatments should consider the aim of care evaluating the risk/benefit assessment. Regarding adjuvant treatments, delays or modifying main treatments may compromise disease control and long-term survival.

In an international position paper, several experts have published some suggestions on the treatment of cancer patients in the COVID-19 era (6). In particular, as ASCO experts, these authors recommended a clear communication and education about hand hygiene, infection control measures, high-risk exposure, and the signs and symptoms of COVID-19. They recommended a strong consideration of the new risk/benefit balance for active intervention, postponing elective surgery, or chemotherapy with low risk of progression analyzing each patient's case.

Another topic of interest should be the future after the Covid-19 crisis ends. Even if the grip of the pandemic loosens, the SARS-CoV-2 could infect other people, especially more brittle patients, such as cancer ones. The return to a normal style of life must be gradual. But for these patients, can it ever be? We will probably have to wait for a vaccine against this virus, as for the flu, with the aim to better protect the health of these patients.

It's a challenging and stressful time for cancer patients under the situation of the worldwide COVID-19 pandemic. In a period of social distancing, cancer patients excluded from outpatient and hospital flows should be brought closer to oncologists through the multimedia means of communication and it should deepen the new chapter of telemedicine. A focus should be placed on the delivery of health-related services and information via media/electronic information. It should allow for oncologists to contact all patients that cannot go to the hospital for monitoring on their health status and suggest care when possible. So far, telemedicine would be considered as a strong *ligand* between patients and oncologists which could guarantee the continuum of care and follow them without losing clinical and human contact. Can we maintain the survival standard by limiting access and therapies? Could we affect the overall quality of life? Or on the contrary, by continuing to administer the same treatment, could we facilitate the spread of the epidemic among these patients by limiting their survival? We believe it is an ethical and moral duty not to abandon these patients in their physical and psychological weakness at this particular historical moment. It remains the task of all investigators to collect data that can be observed, in particular in regards to the possible correlation between immunosuppression and the course of the disease, considering the recent initial evidence of immunosuppressive treatment activities in COVID-19 patients affected by lung complications.

*Last but not least, individual protection measures for all healthcare professionals are clearly essential in order to protect their health as well as that of their patients and family members*.

Probably, this international crisis will lead us to reconsider the *Ars Medica*, which aims to identify the right patient balance between cancer under-treatment and its increase in cancer-related mortality and active cancer treatments potentially linked to a greater risk of death from COVID-19 complications.

## Author Contributions

OB and NS concepted the study. AD, BB, AG, and AR developed the study.

## Conflict of Interest

The authors declare that the research was conducted in the absence of any commercial or financial relationships that could be construed as a potential conflict of interest.

## References

[1] Zhu N Zhang D Wang W Li X Yang B Song J China Novel Coronavirus Investigating and Research Team. A Novel Coronavirus from Patients with Pneumonia in China, 2019. N Engl J Med. (2020) 382:727–33. 10.1056/NEJMoa2001017 31978945 PMC7092803

[2] Wu Y Ho W Huang Y Jin DY Li S Liu SL. SARS-CoV-2 is an appropriate name for the new coronavirus. Lancet. (2020) 395:949–50. 10.1016/S0140-6736(20)30557-2 32151324 PMC7133598

[3] Nelson R Disruptions in Cancer Care in the Era of COVID-19. Available online at: https://www.medscape.com/viewarticle/927215 (accessed March 22, 2020)

[4] Cancer Research UK Available online at: https://www.cancerresearchuk.org/about-cancer/cancer-in-general/coronavirus-and-cancer (accessed March 22, 2020)

[5] https://www.asco.org/asco-coronavirus-information/care-individuals-cancer-during-covid-19 (accessed on 04/07/2020)

[6] Al-Shamsi HO Alhazzani W Alhuraiji A Coomes EA Chemaly RF Almuhanna M. A practical approach to the management of cancer patients during the novel Coronavirus Disease 2019 (COVID-19) Pandemic: An International Collaborative Group. Oncologist. (2020). 10.1634/theoncologist.2020-0213. [Epub ahead of print]. 32243668 PMC7288661

